# Human Factors Engineering Contributions to Infection Prevention and Control

**DOI:** 10.1177/0018720819833214

**Published:** 2019-03-18

**Authors:** Frank A. Drews, Lindsay C. Visnovsky, Jeanmarie Mayer

**Affiliations:** University of Utah, Salt Lake City, USA

**Keywords:** infection prevention and control, personal protective equipment, hand hygiene, bloodstream infections, adherence engineering

## Abstract

**Objective:**

This article provides a review of areas that present significant challenges in infection prevention and control and describes human factors engineering (HFE) approaches that have been applied successfully to these areas. In addition, implications and recommendations for HFE use in future research are discussed.

**Background:**

Infection prevention and control aims to prevent patients and health care personnel from acquiring preventable infections in healthcare. Effective infection control practices of healthcare-associated infections have recently become even more critical with the emergence of life-threatening infections. HFE could benefit infection prevention and control in addressing older and more recent challenges, but uptake has been limited.

**Method/Results:**

This literature review is an integration and synthesis of recently published research that describes HFE-based approaches in infection prevention and control to address the challenges for three specific topics. The results of the review suggests that HFE is in a position to support work in infection prevention and control and improve overall healthcare safety.

**Conclusion:**

HFE provides conceptual frameworks and methods that have significant potential to improve infection prevention and control.

**Application:**

The work reviewed can provide potential solutions for current infection prevention and control challenges by applying HFE based recommendations.

Healthcare as a complex sociotechnical system with many interacting components is well positioned to benefit from human factors engineering (HFE). Among the components are individual factors (people), tasks, technologies/tools, the organization, and the environment (Carayon et al., 2006). Additional elements outside of healthcare settings affect healthcare delivery and include insurance reimbursement policies and regulatory and legal contexts (Vincent et al., 2000).

Previous work used HFE to improve healthcare delivery. However, one specialty that would benefit from increased attention is infection prevention and control. Infection prevention and control is “a practical, evidence-based approach which prevents patients and health workers from being harmed by avoidable infections” (World Health Organization [WHO], 2018). According to WHO, the emergence of life-threatening infections (e.g., severe acute respiratory syndrome, Ebola) emphasizes the importance of effective infection prevention and control practices in healthcare (WHO, 2017). Additionally, the U.S. Department of Health and Human Services (2013) emphasizes preventing the spread of infectious organisms (e.g., severe acute respiratory syndrome, antibiotic-resistant organisms) as a priority for hospitals and healthcare systems. Patients who become colonized in hospitals and then develop symptomatic infections from exposure to organisms such as *Clostridium difficile* or methicillin-resistant *Staphylococcus aureus* drive this focus (Magill et al., 2014; Centers for Disease Control and Prevention, 2016). Finally, emergence of drug-resistant organisms makes infection prevention and control even more critical.

Unfortunately, as pointed out by Jacob, Herwaldt, and Durso (2018), HFE has seen limited application in addressing these challenges to date. Here we provide illustrative examples of infection prevention and control challenges where introduction of HFE models, concepts, and methods was beneficial to identifying solutions. In the next sections, we will focus on HFE contributions to improve hand hygiene, personal protective equipment and central line related activities.

## Improving Hand Hygiene Adherence

Annually, healthcare-associated infections affect 1.7 million patients in the United States, causing nearly 100,000 deaths (Klevens et al., 2007). An important strategy for preventing healthcare-associated infections is healthcare personnel (HCP) performing proper hand hygiene. Hand hygiene adherence is low, estimated to average 40% among HCP (WHO, 2009), and even small increases in adherence substantially reduce infections (WHO, 2017). However, the success and sustainability of current interventions to improve hand hygiene adherence varies. Hand hygiene is located at the interface between human behavior and technology (e.g., dispensers) and HFE can contribute the theoretical depth and practical knowledge necessary for developing interventions.

Work by Sax et al. (2007) applies a human factors design approach and conceptualizes proper hand hygiene as following the five moments at which transmission of an infectious organism could occur (Sax et al., 2009). The five moments are before patient contact; before performing an aseptic task; after exposure to body fluids; after patient contact; and after contact with the patient’s surroundings. Unfortunately, hand hygiene adherence following the five moments is low (i.e., 42%; Scheithauer, Batzer, Dangel, Passweg, & Widmer, 2017).

One perspective to explain low hand hygiene adherence is to view it as an issue of training deficits. However, although training deficits are part of a comprehensive explanation for nonadherence, a sociotechnical systems perspective may provide alternative explanations and help develop successful and sustainable interventions. For example, by using mental model deficits as an explanation, Sax and Clack (2015) argue that inaccurate mental models may lead to breakdowns in hand hygiene and that experience and alignment of the models with the workplace may lead to their optimization. Thus, HFE-based workplace modification that increases hand hygiene saliency—such as purposeful placement of hand sanitizer dispensers in convenient, noticeable locations—may increase adherence.

Cure and Van Enk (2015) examined the effect of hand sanitizer dispenser usability on hand hygiene adherence. Usability included visibility and proximity to room entrance and point of care, easy and unobstructed access, location along the physical workflow path, and dispenser installation height. In addition, standardization, i.e., uniformity of dispenser placement across unit(s), was assessed. The results suggest that dispenser visibility and proximity to entrance increased adherence, whereas standardization did not.

Patterson et al. (2014) identified issues preventing hand hygiene adherence, among them inconsistent location of hand sanitizer dispensers, separation of the glove storage location from the hand sanitizer dispenser, and sink use for storage, rendering it unusable for hand washing. In this study, nurses also emphasized the importance of making hand sanitizer dispenser locations more salient. This is consistent with work demonstrating that drawing attention to dispensers with blinking LED lights increases hand hygiene adherence (Nevo et al., 2010). Another workplace modification is to increase the saliency of the hand hygiene requirement by demarcating the space around a patient by placing markers or tape on the floor (Muder et al., 2008).

Other research has concluded that multimodal interventions, a common strategy in infection prevention and control, where several strategies and intervention elements are utilized simultaneously to target multiple psychological aspects, are more effective than singular interventions (Huis et al., 2012; Naikoba & Hayward, 2001; Pincock, Bernstein, Warthman, & Holst, 2012). WHO guidelines for a multimodal intervention (WHO, 2009) recommend changes in five areas: system level, education and training, evaluation and feedback, reminders in the workplace, and institutional safety climate.

A recent systematic qualitative literature review (Smiddy, O’Connell, & Creedon, 2015) identified issues affecting HCPs’ compliance with hand hygiene guidelines. The results align well with a sociotechnical perspective on hand hygiene adherence that differentiates individual factors, tasks, technologies, and the organization as affecting performance.

To summarize, hand hygiene adherence can be improved by carefully considering dispenser usability, workflow, and other aspects of the sociotechnical system. In the same vein, HCP mental models need to be better understood and their accuracy improved. Future HFE work can increase our understanding of how to improve hand hygiene adherence further.

## Use of Personal Protective Equipment (PPE)

PPE protects HCP from potential exposure to contaminated body fluids as well as from exposure to organisms transmitted via contact, droplet, or airborne routes. The importance of proper PPE use is illustrated by two potentially fatal viral infections, Ebola virus and severe acute respiratory syndrome–associated coronavirus. The West African Ebola outbreak in 2014–2016 resulted in more than 28,000 infected patients and 11,000 fatalities (WHO, 2016). Similarly, severe acute respiratory syndrome-associated coronavirus resulted in an outbreak during the early 2000s. Both outbreaks highlight the importance of proper PPE use, as many HCP were infected in each due to poor PPE doffing (removal) practices (CDC, 2003).

Correct PPE use intersects equipment, user, and environments (physical, social, and policy). Three phases of PPE use can lead to potential contamination: donning, patient care, and doffing. During donning, contamination risks are associated with improper storage of the PPE, previous PPE exposure to germs, or incorrect technique. During patient care, risks include damaging the PPE, design flaws, and sidestepping PPE due to distraction (i.e., reaching for a cell phone under the PPE). Doffing risk is due to incorrect PPE removal, incorrect handling and disposal of PPE, or damaging PPE. Below, we will review the literature on PPE donning and doffing practices and PPE-related design efforts.

### PPE Donning and Doffing

Puro and Nicastri (2004) reported that HCP in the severe acute respiratory syndrome outbreak lacked understanding of proper PPE removal. In addition, they found that literature and guidelines lacked necessary detail and even provided ambiguous or contradictory removal recommendations, resulting in contamination risk.

Beam, Gibbs, Boulter, Beckerdite, and Smith (2011) assessed PPE use for common pathogens (airborne and contact isolation precautions) in a simulator study. Participants performed care tasks, and contamination was measured using fluorescent marker. All of the participants committed at least one PPE breach, with breaches occurring during all three phases. Donning issues included failure to perform a respirator seal check, failure to tie the gown at the neck and waist, and improper donning sequence. Doffing breaches included deviations from correct doffing sequence and improper mask removal. In addition, HCPs brought out potentially contaminated items during room exit. Common breaches during care included touching HCPs unprotected body areas with contaminated PPE. Overall, the authors concluded that HCP understanding of contamination pathways needed improvement and simulator studies help gain insights into nonadherence.

Similarly, Zellmer, Van Hoof, and Safdar (2015) in an observational, nonsimulation study found 57% of HCP doffed PPE in an incorrect order, 53% failed to remove contaminated PPE before room exit, and 40% disposed of PPE incorrectly. The authors concluded that deviations from protocol were common. This conclusion is also supported by Mitchell et al. (2013), who used a standardized data collection tool to record the initial choice and later removal of PPE. The authors found that 66% of all HCP failed to don the recommended PPE, and 56% doffed in an incorrect sequence.

Mumma et al. (2018) performed a human factors risk analysis of doffing practices for Ebola-level PPE to assess the relationship between errors and self-contamination. In their simulator study of 11 highly experienced HCPs that included a failure modes and effects analysis, they found that hand hygiene and removal of the powered air-purifying respirator contributed most to infection risk and errors. The authors emphasize that PPE can protect but also endanger HCPs, highlighting the importance of equipment design that reduces the potential for endangering HCPs.

By applying an HFE analytical framework, Krein et al. (2018) conducted a qualitative observational study of 325 isolation precaution rooms in multiple hospitals and units to identify and characterize failures in PPE use. Observers recorded field notes of HCP providing patient care, focusing on self-contamination events. Of the 283 observed failures, 102 were categorized as violations, 144 as process/procedural mistakes, and 37 as slips. Examples for these failures were entering the room without required PPE (violation), PPE doffing sequence errors (mistake), and wiping one’s face with potentially contaminated, gowned forearms (slip). The authors concluded that given the range of contributors to self-contamination events, no single strategy is sufficient to reduce transmission risk.

This conclusion is supported by an earlier study by Casanova, Alfano-Sobsey, Rutala, Weber, and Sobsey (2008), which suggested that even reviewing doffing protocol before PPE removal and instruction availability during doffing were still associated with a significant number of mistakes. Similarly, Kang et al. (2017) evaluated two PPE types, simple or full-body sets, and assessed contamination using fluorescent powder. In their simulator study, they observed contamination in 92% of doffing attempts with simple, and in 66% with full-body PPE. After receiving training, 91% of HCP still experienced contamination during doffing in the follow-up simulation. The authors pessimistically concluded that HCP contamination during doffing “seems to be caused by natural flaws” (Kang et al., 2017, p. 22), which ignores the contribution that better PPE design could have on performance.

A more optimistic view of the effect of training is described by Casalino et al. (2015) who evaluated the effect of two training programs (conventional and reinforced) on use of basic or enhanced PPE. Both programs provided students with theoretical training, with instructors announcing and reinforcing donning and doffing steps during practice sessions. The authors reported reduced contamination over three training sessions for both training methods, but reinforced training achieved higher adherence with both PPE types. However, performance was never optimal: even after all sessions, nonadherence to best practices was still observed.

Training on PPE use continues to be challenging. Published guidelines are often ambiguous or underspecified, and protocols differ by PPE product (e.g., whether gowns are tied or donned over-the-head). Additionally, it is unclear what the most effective and efficient training methods are and whether feedback and proficiency demonstrations should be required. Given that adequate training is time- and resource-intensive, requiring tracking of completion and proficiency, it is unsurprising that training may not always be available. Overall, this view is consistent with the perspective that classifies training as “weaker” than other interventions (Bagian, King, Mills, & McKnight, 2011).

John et al. (2016) surveyed HCP perspectives on PPE training and identified suboptimal training and lack of proficiency testing as contributors to failures during glove and gown removal. In addition, 15% of physicians reported not having received PPE training, compared with 1.4% of nurses. Physicians reported receiving annual training 12% of the time and nurses 47%; on-the-job training was the most common means of training delivery (47.3%). Participants also received a mandatory annual computerized infection prevention training; however, only 43% of surveyed HCP recalled participation, illustrating the training’s limited impact.

One way to provide effective training is by delivering immediate performance feedback. Studying gown and glove removal with a fluorescent marker, Tomas et al. (2015) found that contamination was common (46% of the cases) and higher with glove removal. Also, contamination was higher during lapses in proper technique. Training involving immediate feedback during PPE doffing contamination events reduced skin and clothing contamination during subsequent glove and gown removal. Interestingly, the authors demonstrated intervention sustainability in 1- and 3-month follow-ups.

Most recently, in response to the Ebola outbreak, the CDC funded work to develop a web-based training program to complement and clarify PPE guidelines (Gurses, Rosen, & Pronovost, 2018). The researchers applied HFE principles and methods to translate PPE use guidelines into instructions focused on reduced guideline ambiguity, redesigning the work system, and improving teamwork of HCP caring for Ebola patients. Unfortunately, effectiveness of the program still needs evaluation.

### PPE-Related Redesign Efforts

At this point, little work focused on the redesign of PPE to make it more user-friendly. Singleton and Johnson (2018) developed an HFE-based storage cart for PPE and evaluated PPE compliance before and after its introduction. Cart design elements included standardization, picture labels, and PPE use instructions. Baseline PPE compliance was only 47%, but increased to 81% after cart introduction.

DuBose et al. (2018) focused on the built environment and had 41 HCP perform donning and doffing under instruction of trained observers in four Ebola treatment centers and one high-fidelity mock-up. They identified limitations in communication between observers and HCP and visibility of HCP. Size and layout of the doffing area also affected adherence; lack of demarcation lines for the contaminated zone contributed to HCP confusion.

Taken together, PPE-related issues are well documented and, due to the complex nature of the equipment and procedures required for correct PPE use, there are many challenges. Issues relate to clear communication on the type of PPE required and how to don and doff this equipment. Moreover, although there are needs for additional, effective training, failure to require PPE use training is a significant organizational issue. Given relatively low adherence rates, HFE has great potential to increase adherence via improved training, equipment, and built environments. Given that low adherence must be addressed from a number of angles, a sociotechnical systems perspective appears to be the way to integrate the separate efforts into one comprehensive, effective approach.

## Reducing Central Line Associated Blood Stream Infections

More than 400,000 intensive care patients acquire a healthcare-associated infection each year, and bloodstream infections account for the highest number of deaths (Januel et al., 2010; Klevens et al., 2007; Zimlichman et al., 2013). Over the past decade, significant efforts have resulted in an 80% reduction in central line associated bloodstream infections (CLABSI) in U.S. intensive care units (Pronovost, Cleeman, Wright, & Srinivasan, 2016). This decrease in CLABSI is partly attributable to interventions similar to those implemented in a collaborative of 103 intensive care units (Michigan Keystone project; Pronovost et al., 2008). The intervention targeted HCP behavior changes and included both technical and organizational components (Dixon-Woods, Bosk, Aveling, Goeschel, & Pronovost, 2011). Participating hospitals first addressed organizational issues by having site leaders implement a comprehensive unit-based safety program to improve safety culture. The program trained staff in hazard recognition, safety culture, teamwork, and communication. The specific bundle of interventions to reduce CLABSI included education, providing central line carts with needed supplies, a checklist of practices for safe line insertions, permission to stop HCP if practices were not adhered to, and daily central line removal discussions. An implementation in Spain that initially focused on the use of checklists alone did not reduce CLABSI; only implementation of the full program led to a reduction (Palomar et al., 2013), emphasizing the importance of a sociotechnical systems perspective.

Line maintenance issues are another CLABSI contributor. The conceptual framework of adherence engineering addresses increasing adherence to protocols in complex tasks (Drews, 2013). The core assumption of adherence engineering is that task behavior is partly shaped externally and that external factors can be used to increase protocol adherence. Seven principles aim at increasing adherence: (1) deliberately create object affordance (a quality of an object/environment that promotes performance of a specific action), (2) provide task-intrinsic guidance (the task guides the user on how to perform it), (3) implement nudging (provide optimized choices), (4) select and implement smart defaults (provide default values that are commonly used), (5) provide feedback, and (6) reduce the cognitive effort required for a task, and (7) reduce the physical effort required for a task.

An HFE-based central line maintenance kit was developed by applying this framework (Drews, Bakdash, & Gleed, 2017). Table 1 lists the adherence engineering principles, the goals, and the implementation in the kit. Clinical performance using the central line maintenance kit was evaluated in clinical observations. This 29-month, pre-post implementation assessment study suggests that the HFE-based kit improved adherence and lead to a significant reduction in the number of CLABSI. Thus, application of HFE design principles to medical kit development can improve protocol adherence and clinical outcomes.

**Table 1:** Adherence Engineering Principles, Goals, and Kit Implementation Examples

Principle	Goal	Implementation
Affordances	Make use intuitive	Tabs to open kit, visibility of flaps of pockets
Task intrinsic guidance	Provide structure/preview of task sequence	Sequential order of pockets, when multiple items then added information about sequence of use
Nudging	Support adherence by suggesting desirable actions	Providing hand gel in pockets, providing pen to remind to date the dressing
Smart defaults	Help select desirable actions/ material	Selection of contents that follow best practices
Feedback	Facilitate resumption and performance assessment	Pockets are visibly empty after step completion supporting resumption
Minimizing cognitive effort	Support task execution by reducing cognitive demand	Chunking of related activities, icons, and labels as reminders, structured sequence, reduction in planning needs for procedure
Minimizing physical effort	Make adherence convenient	Reduction of walking requirements (e.g., to hand gel dispenser, to supplies room to pick up missing items)

In summary, successful interventions targeted HCP behavior with a focus on technical and organizational components. The development of better tools (carts, kits) improves performance, as does training in hazard recognition, teamwork, communication, and a culture of safety. Finally, by developing conceptual frameworks, guidance can be provided to develop future interventions that support HCPs in their tasks.

## Conclusions

HFE has been applied to a limited extent in infection prevention and control. A sociotechnical systems perspective that is essential to the HFE approach has significant potential to improve HCP performance and patient safety. HFE frameworks have widespread applicability for guiding future interventions. This review suggests that HFE can contribute in numerous ways to improving performance at the individual, group, and system level. Example areas of contribution are (1) development/application of conceptual frameworks of human performance, (2) improved understanding of HCP cognitive processes (e.g., individual and shared mental models), (3) simplification or redesign of workflows, (4) improvement of equipment design, (5) development/optimization of standardized training programs and requirements, (6) elimination of communication/guidelines ambiguity, (7) task-based improvements of the built environment and standardization of equipment placement within and across facilities, and (8) improvements of organizational safety climate. Table 2 provides a selective overview of research topics, areas of contribution, implications of the research, and references reviewed above.

**Table 2:** Research Topics, Contributions, and Implications of HFE Research in Infection Prevention and Control

Topic	Contribution (Area)	Implications	References
Hand hygiene	Identification of relationship between spatial layout and HH adherence (7)	Optimization of dispenser visibility and location	Cure & Van Enk (2015)
	Importance of mental models for task performance (2)	Analysis and design of tasks to address user mental models	Sax & Clack (2015)
PPE	Design of PPE carts (4)	Optimization of equipment	Singleton & Johnson (2018)
	Categorization of PPE breakdowns using HFE framework (1)	Importance of HFE frameworks to analyze performance failures	Krein et al. (2018)
	Development of PPE training (6)	Importance of systematic training development	Gurses et al. (2018)
	Identification of communication issues. Spatial layout affected adherence; lack of demarcation lines contributed to HCP confusion (6)(7)	Spatial layout and communication improve performance	Dubose et al. (2018)
Central lines	Development and evaluation of Michigan Keystone Project, reducing CLABSI (4)(7)(8)	Importance of individual, team and organizational factors; develop and provide standardized equipment (carts)	Pronovost et al. (2008)
	Development and evaluation of maintenance kit to improve procedural adherence and reduce CLABSI (1)(3)(4)	Development and application of HFE framework to kit design, development, and evaluation	Drews et al. (2017)

*Note.* HH = hand hygiene; PPE = personal protective equipment; HFE = human factors engineering; HCP = health care professional; CLABSI = central line associated bloodstream infection.

The application of HFE has successfully improved performance and safety in numerous domains. Today, HFE is in a position to contribute to infection prevention and control to the benefit of those who provide care, and those who receive care.

## Key Points

- Application of a sociotechnical systems perspective HFE can help improve infection prevention and control.
- HFE based improvements have been made in the areas of personal protective equipment use, hand hygiene, and central line associated bloodstream infections.
- There is a clear need for future research to improve infection prevention and control applying human factors engineering
